# Enhanced Sleep Is an Evolutionarily Adaptive Response to Starvation Stress in *Drosophila*

**DOI:** 10.1371/journal.pone.0131275

**Published:** 2015-07-06

**Authors:** Melissa E. Slocumb, Josue M. Regalado, Masato Yoshizawa, Greg G. Neely, Pavel Masek, Allen G. Gibbs, Alex C. Keene

**Affiliations:** 1 Department of Biology, University of Nevada-Reno, Reno, NV, 89557, United States of America; 2 School of Life Science, University of Nevada-Las Vegas, Las Vegas, NV, 89119, United States of America; 3 Department of Biology, University of Hawai’i, Manoa, 96822, United States of America; 4 Neuroscience Division, Garvan Institution, Sydney, NSW 2010, Australia; Imperial College London, UNITED KINGDOM

## Abstract

Animals maximize fitness by modulating sleep and foraging strategies in response to changes in nutrient availability. Wild populations of the fruit fly, *Drosophila melanogaster*, display highly variable levels of starvation and desiccation resistance that differ in accordance with geographic location, nutrient availability, and evolutionary history. Further, flies potently modulate sleep in response to changes in food availability, and selection for starvation resistance enhances sleep, revealing strong genetic relationships between sleep and nutrient availability. To determine the genetic and evolutionary relationship between sleep and nutrient deprivation, we assessed sleep in flies selected for desiccation or starvation resistance. While starvation resistant flies have higher levels of triglycerides, desiccation resistant flies have enhanced glycogen stores, indicative of distinct physiological adaptations to food or water scarcity. Strikingly, selection for starvation resistance, but not desiccation resistance, leads to increased sleep, indicating that enhanced sleep is not a generalized consequence of higher energy stores. Thermotolerance is not altered in starvation or desiccation resistant flies, providing further evidence for context-specific adaptation to environmental stressors. F_2_ hybrid flies were generated by crossing starvation selected flies with desiccation selected flies, and the relationship between nutrient deprivation and sleep was examined. Hybrids exhibit a positive correlation between starvation resistance and sleep, while no interaction was detected between desiccation resistance and sleep, revealing that prolonged sleep provides an adaptive response to starvation stress. Therefore, these findings demonstrate context-specific evolution of enhanced sleep in response to chronic food deprivation, and provide a model for understanding the evolutionary relationship between sleep and nutrient availability.

## Introduction

Sleep is a near universal animal behavior, with highly conserved functional and molecular properties [1,2]. Sleep duration and timing vary greatly between species in accordance with ecological, environmental, and evolutionary history [3,4]. Animals modulate sleep in response to a number of factors, including environmental stressors, developmental stage, and aging [5–7]. While sleep is clearly influenced by many environmental factors, sleep timing and duration are closely related to nutrient availability and foraging strategy [8,9]. Both flies and mammals suppress sleep in response to starvation, presumably in order to forage for food. This indicates a functional trade-off between sleep duration and feeding [7,10,11]. Conversely, one proposed function of sleep is energy conservation, suggesting prolonged sleep may improve survival in the absence of nutrients [12]. Although there are likely evolutionary interactions between sleep and nutrient availability, these interactions are not well understood.

The fruit fly *Drosophila melanogaster* presents a powerful model for investigating genetic interactions between sleep and metabolic processes [13,14]. Resistance to nutrient deprivation is associated with enhanced metabolic stores, as well as physiological or behavioral adaptations that conserve energy [15–17]. Water and food represent two primary nutrient sources, and *Drosophila* appear to have developed distinct mechanisms to cope with the deprivation of each nutrient source. Starvation and desiccation resistance in wild populations of *Drosophila* have been studied extensively across many geographic ranges and are often found to be strongly correlated with the lipid or glycogen content of the flies [15,18,19]. Many traits associated with stress resistance vary greatly due to naturally occurring genetic variation, providing the opportunity to identify genetic regulators of these traits. Genomic analyses of fully sequenced inbred lines and quantitative genetic approaches have provided insight into the genetic basis for resistance to environmental and physiological stress [20,21]. While these studies have provided insight into the molecular underpinnings of many traits related to stress resistance, the functional and evolutionary interactions between sleep and nutrient deprivation remains unclear. Here, we examine the evolutionary relationship between sleep duration resistance to food and water deprivation.

Experimental evolution in wild-caught *Drosophila melanogaster* provides a powerful approach to study the evolutionary basis for, and interaction between, traits [22, 23]. Previous work has demonstrated changes in sleep and activity in flies selected for starvation resistance [24], but it is not clear whether these represent generalized adaptations to stress or selective changes to prolong survival in response to starvation. We have utilized experimental evolution to generate flies with enhanced resistance to starvation and desiccation, providing the opportunity to examine the evolutionary and functional relationship between these traits. Three populations of wild-caught flies were independently selected over 60 generations under conditions of starvation resistance (SR) or desiccation resistance (DR), allowing for the examination of repeated evolutionary changes in response to distinct forms of nutrient stress [23,24]. Flies selected for starvation resistance survive up to 18 days in the absence of food, while non-selected controls survive an average of four days [23,24]. Selection for desiccation resistance results in flies that survive up to 4 days in the absence of water, nearly twice the survival time of non-selected controls [23,24]. Here, we examine the sleep and activity phenotypes of flies selected for SR and DR to determine whether conserved or distinct changes in activity contribute to the generation of resistance to starvation and desiccation.

Both energy stores and resistance to nutrient deprivation differ between flies selected for DR and SR, suggesting that independent genetic mechanisms regulate evolutionary changes that result from chronic nutrient deprivation. Selection for SR, but not DR, results in flies with prolonged sleep, suggesting that change in sleep is not a generalized response to environmental stress. F_2_ hybrids generated from SR-DR selected flies display an interaction between sleep and starvation resistance, but not sleep and desiccation resistance, supporting the notion that prolonged sleep duration is an evolutionarily adaptive response to surviving starvation stress, specifically. These findings provide evidence for the context-dependent evolution of metabolic and behavioral adaptations in response to nutrient deprivation and introduce a framework for understanding the evolutionary basis for interactions between sleep and food availability.

## Materials and Methods

### Generation of starvation and desiccation resistant flies

**Fig 1:**
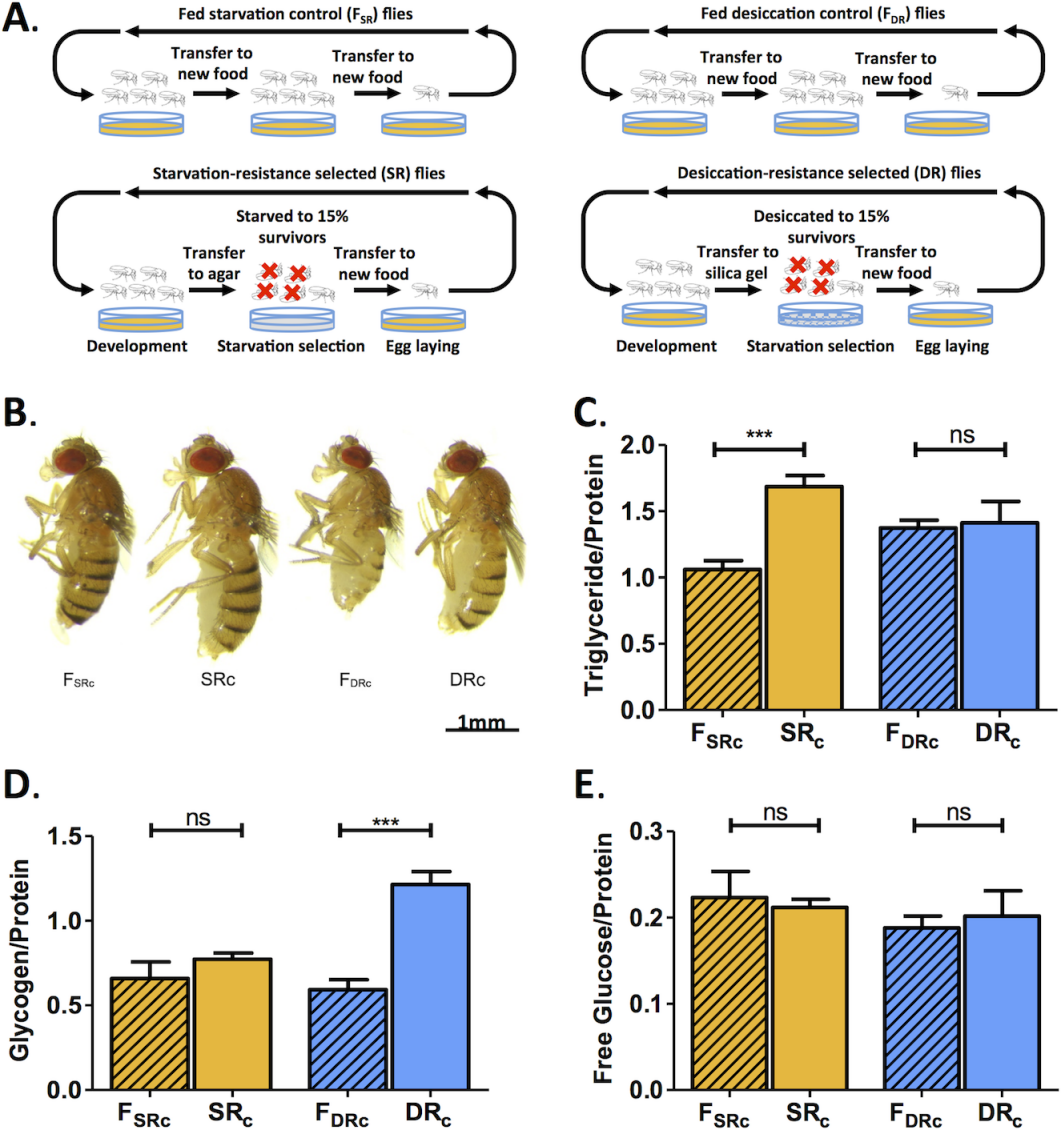
SR and DR selection increases body size and alters metabolic profile. A) Schematic of selection processes for SR and DR flies. Adult outbred flies were placed under desiccation or starvation conditions until ~15% of the flies survived. The flies were then moved to food. This process was repeated over >80 generations. The F_DR_ controls were placed on agar during desiccation selection to account for food deprivation in DR selected flies. There were three replicated SR populations (designated SR_a_, SR_b_ and SR_c_) and three fed control populations (F_SRa,_ F_SRb_ and F_SRc_). For DR experiments there were three replicated groups (designated DR_a_, DR_b_ and DR_c_) and three fed control populations (F_DRa,_ F_DRb_ and F_DRc_). B) Flies from the C Group. SR_c_ and DR_c_ flies are visibly larger than F_SRc_ and F_DRc_ controls. C) Triglyceride levels are elevated in the SR_c_ flies compared to F_SRc_ controls. No differences are observed between DR_c_ flies and F_DRc_ controls (P<0.001; See S1 Table). D) Glycogen levels were greater in DR_c_ flies than in F_DRc_ controls. No differences were present between SR_c_ flies and F_SRc_ controls (P<0.001; See S1 Table). E) Free glucose levels did not differ between SR_c_ or DR_c_ flies and their respective controls (P>0.05; See S1 Table).

The wild-derived stocks used in this study were collected with owner’s permission from Terhune Orchards in Princeton, N.J. in 1999 and have been maintained as outbred stocks at 25°C on standard corn meal medium since this time. The generation of DR and fed control F_DR_ flies have previously been described as Td and Tf flies, respectively ([23] and Fig 1B). These have been renamed in this manuscript for clarity, with the three replicated DR populations being designated as DR_a_, DR_b_, and DR_c_, and the three fed control populations as F_DRa_, F_DRb_, and F_DRc_. Briefly, these three populations of DR flies were selected from the founding stock that had been maintained on standard food conditions. Selection for desiccation resistance in DR flies occurred by transferring populations of ~7,500 adult flies to a population cage containing silica gel desiccant alone. The silica gel was replaced with fly food when ~15% of the flies survived. Eggs were then collected from the progeny, and this cycle was repeated for 30 generations to develop the previously described DR lines. The DR lines used in this paper have been maintained under reduced desiccation selection (24 hours under desiccation for each generation) for ~110 generations. The F_DR_ flies used in this paper were three replicate fed control populations maintained on food throughout the selection process (Fig 1B). Because desiccation selection involves removal of both food and water, an additional population of lines was generated where food deprivation was yoked to the desiccation selected *Drosophila*. These flies were provided agar instead of the silica gel desiccant, and flies were transferred at the same times as DR group flies [23]. Of the three groups originally generated, only two remain, and these flies have been renamed DR_CTRLa_ and DR_CTRLb_ for clarity.

The SR and fed control F_SR_ populations were derived from two control treatments for desiccation-selected populations described in [23]. These SR lines and controls were previously described in [24]. For the selection process, approximately 8,000 experimental flies for each of the three starvation selected groups were maintained in constant light at room temperature (~23°C) on 1% agar until only 15–20% of the original population survived. Surviving flies were then placed on food to lay eggs. The next generation of adults was selected for starvation resistance in the same manner. Flies assayed for behavior experiments described in this manuscript ranged between generations 55 and 70 of selection. F_SR_ populations were maintained on food while the SR populations were starved. There were three replicated SR populations (designated SR_a_, SR_b_, and SR_c_) and three fed control populations (F_SRa_, F_SRb_, and F_SRc_). It should be noted that fed control populations are also demographic controls, as they are maintained in the same generation time as their associated stress-selected populations. All selection occurred in the laboratory of Allen Gibbs (UNLV).

### *Drosophila* maintenance

Flies taken off of the selection process for behavioral experiments were maintained and tested in humidified incubators at 25°C and 65% humidity (Powers Scientific). Flies were reared on a 12:12 light-dark cycle for 2–6 generations following selection prior to behavioral analysis. All flies were maintained on Jazz-Mix *Drosophila* Food (Fisher Scientific).

### Protein, glycogen, and triglyceride measurements

For quantifying triglyceride, glycogen and protein content of flies two female flies aged 3–5 days were homogenized in HCl, pH 7.4, 0.1% Triton-X, 1X protease inhibitor cocktail (Sigma Aldrich, P8340). Triglyceride concentration was measured using the Stanbio Liquicolor Kit (Boerne, TX, S2200), and protein concentrations were measuring using a BCA Protein Assay Kit (Pierce Scientific, 23225). Total glucose levels were determined using the Glucose Oxidase Reagent (Pointe Scientific, G7519) in samples previously treated with 8 mg/mL 48 amyloglucosidase in 0.2M Sodium Citrate buffer, pH 5.0 (Boston BioProducts, BB-88). Free glucose was measured in samples not treated with amyloglucosidase, and then glycogen concentrations were determined by subtracting the free glucose from total glucose concentrations. Both glycogen and triglyceride concentrations were standardized to the total protein content of each sample containing two flies.

### Sleep and activity analysis

#### Activity monitoring using *Drosophila* Activity Monitoring (DAM) System

Fly activity was monitored using DAM2 *Drosophila* activity monitors (Trikinetics, Waltham, MA) as previously described [25,26]. Female flies were briefly anesthetized using CO_2_ within 1 hour of lights on at Zeitgeber Time 0 (ZT0) and placed into plastic tubes containing standard food. The DAM system measures activity by detecting infrared beam crossings for each animal. These data were used to calculate sleep information by extracting immobility bouts of 5 minutes or more using the *Drosophila* Sleep Counting Macro [27]. Multiple variables of sleep were analyzed, including total sleep duration, sleep bout number, and average sleep bout length as previously described [27,28]. For experiments examining the effects of starvation on sleep, activity was recorded for one day on food prior to transferring flies into tubes containing 1% agar (Fisher Scientific). Flies were then transferred every 7 days onto fresh agar tubes for the remainder of the experiment. For experiments examining the effects of desiccation on sleep, activity was recorded for one day on food prior to transferring flies into tubes containing dry Kimwipes (Fisher Scientific).

### Stress survival

Flies subjected to stress survival tests were first acclimated in DAM2 monitor tubes containing standard fly food for 24 hours. For experiments examining the effects of stress on longevity, flies were then transferred into individual DAM2 tubes and were assayed under starvation, desiccation, or heat shock conditions. A 1% agar (Fisher Scientific) solution was made to replicate starvation selection conditions; kimwipes were used to represent desiccation conditions; and a temperature increase to 35°C was used to generate heat stress conditions. Activity was recorded in DAM2 monitors and measured using the Sleep Counting Macro [27]. Death was manually determined at the last activity time point from the final recorded activity bout for each individual fly. For analysis, we applied Kaplan-Meier analysis by grouping each stress resistant population to its respective control.

### Statistics

Statistical analyses were performed using InStat software (GraphPad Software 5.0 Inc.) or IBM SPSS 22.0 software (IBM, Somers, NY, USA). We employed two-way ANOVA for most of the comparative analyses, followed by posthoc analysis if necessary. In the slope analysis, we used ANOVA to compare the slopes of grouped F_SR_ (F_SRa_, F_SRb_, and F_SRc_) and SR (SR_a_, SR_b_, and SR_c_) populations. In the figures, graph bars are mean values and error bars represent the standard error of the mean. All statistics are fully reported in S1 Table.

## Results

### Altered energy stores in starvation and desiccation resistant flies

Three independent groups of flies were derived for starvation (SR) or desiccation (DR) resistance from a previously described outbred population [23, 24]. SR flies were generated by maintaining flies on agar until ~15% of flies survived, while fed control flies (F_SR_) were maintained on food (Fig 1A). DR selected flies were maintained on silica gel desiccant until ~15% survived, while fed control flies (F_DR_) were maintained on food [23] (Fig 1A). Consistent with previous reports, both SR and DR selection resulted in increased body size compared to F_SR_ and F_DR_ group controls for all three replicates tested ([23,24] Fig 1B and data not shown). Triglyceride and glycogen represent the primary energy stores in *Drosophila*. Triglycerides provide a more efficient method of energy storage, while glycogen provides a source of metabolic water. This raises the possibility that the two selection processes result in a differential accumulation of these energy stores [16]. We found that triglyceride levels are significantly higher in all three groups of SR flies compared to F_SR_ control flies, while no difference was observed between DR selected flies and F_DR_ controls, indicating that only selection for starvation resistance results in increased triglyceride accumulation (Fig 1C and S1 Fig). Glycogen levels are elevated in DR flies in all groups compared to F_DR_ control flies, while no differences are observed between SR and F_SR_ controls in Groups B and C. However, SR flies in Group A do have increased glycogen levels, along with their increase in triglyceride levels (Fig 1D and S1 Fig). No significant differences in free glucose were observed between any of the lines tested. However, for Group A, free glucose is elevated in SR and reduced in DR selected groups compared to controls, indicating that selection primarily effects triglyceride energy stores in this group (Fig 1E and S1 Fig). These findings reveal distinct differences in physiological phenotypes between independently selected DR and SR lines. Therefore, selection for starvation resistance results in enhanced triglyceride levels, while selection for desiccation resistance results in increased glycogen stores. These findings suggest that distinct metabolic phenotypes are associated with the evolution of resistance to starvation and desiccation stress.

### Selection for starvation and desiccation resistance has differential effects in response to nutrient deprivation

**Fig 2:**
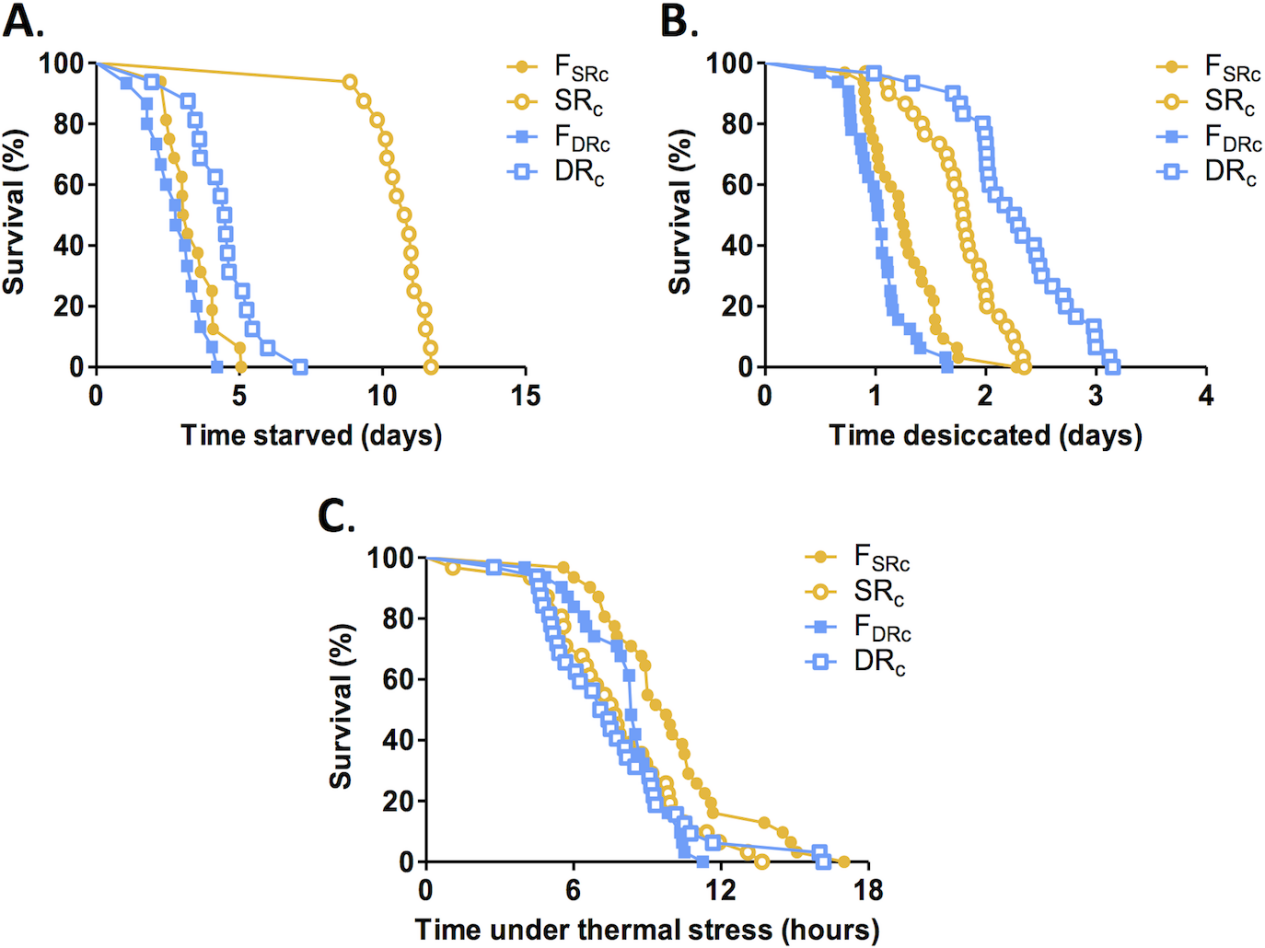
Distinct resistance to nutrient deprivation in SR and DR flies. Survival of flies placed in activity monitors under starvation conditions. A) Flies from the SR_c_ lines survived longer than F_SRc_ controls, whereas DR lines do not differ from F_DRc_ controls (SR lines: P < 0.001 in all groups; DR lines: P>0.05). B) DR_c_ flies survive longer than F_DRc_ controls under desiccation conditions. SR_c_ flies were also resistant to desiccation compared to F_SRc_ controls (DR_c_ line: P<0.001; SR_c_ line: P<0.001, See S1 Table.) C) SR_c_ flies did not live as long as F_SRc_ controls, and no difference in longevity was observed in DR_c_ flies and controls, under thermal stress conditions (SR lines: P = 0.01; DR lines P>0.05; See S1 Table).

To determine whether each selection protocols generally enhanced stress resistance or increased survival to nutrient deprivation in a context-dependent fashion we measured longevity of SR and DR selected flies under starvation and desiccation conditions. Following 24hrs of acclimation on food, flies were transferred to tubes containing 1% agar or dry Kimwipes. Survival time was measured using the Drosophila Activity Monitor (DAM) system [29]. Under starvation conditions, all three SR groups survived longer than F_SR_ and F_DR_ controls and DR flies (Fig 2A and S2 Fig). However, two groups of desiccation selected DR flies survived longer than associated controls, suggesting that selection for desiccation resistance may confer moderate starvation resistance (Fig 2A and S2 Fig). Under desiccation conditions, all three groups of SR flies survived longer than F_SR_ group controls, and all three groups of desiccation selected DR flies survived longer than F_DR_ controls (Fig 2B and S2 Fig). Therefore, experimental selection for starvation or desiccation resistance has differential effects on the evolution of resistance to nutrient deprivation.

It is possible that SR and DR selection results in flies that are selectively resistant to nutrient deprivation, or that are generally resistant to stress. Previous reports indicate that DR selected flies showed a generalized resistance to stressors, including chemical, heat, and radiation stressors [16,17]. To differentiate between these possibilities, the longevity of SR and DR flies under conditions of high-temperature stress was assessed. Flies were maintained at 35°C, and longevity was measured. No increased longevity was observed between SR or DR flies and their controls, suggesting that their enhanced survival in response to nutrient deprivation is not generalizable to other stressors (Fig 2C and S2 Fig). Therefore, the enhanced resistance to nutritional deprivation following the selection protocol used to generate the flies in this study does not result from generalized stress resistance.

### Sleep is not altered in flies selected for desiccation resistance

**Fig 3:**
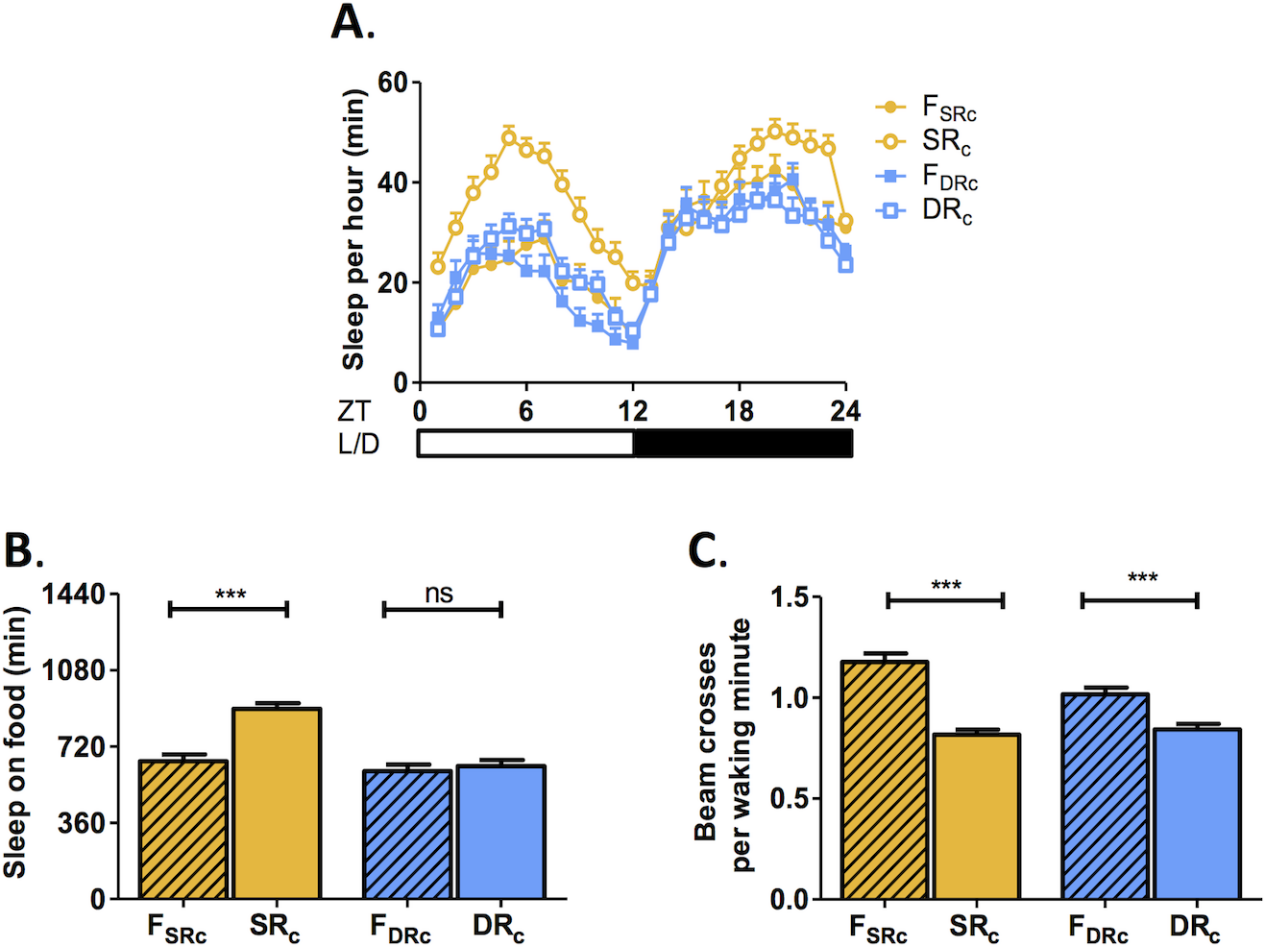
Selection for DR does not alter sleep. A) Sleep profiles depicting hourly sleep reveal that sleep in SR_c_ flies is increased during both day and night compared to the DR_c_ flies and respective controls (N = 64 for all groups). B) The total sleep duration over 24hrs on food is significantly longer in SR_c_ flies than in F_SRc_ flies. No differences are observed between DR_c_ flies and F_DRc_ flies (SR_c_ group: P<0.001; DR_c_ lines: P>0.05; See S1 Table). C) Beam crossings per waking minute are reduced in DR_c_ and SR_c_ flies compared to respective controls (SR_c_ group: P<0.001; DR_c_ lines: P<0.001; See S1 Table).

SR flies sleep longer than their controls, raising the possibility that prolonged sleep is adaptive for survival under conditions of chronic nutrient deprivation [24]. It is possible that the evolution of prolonged sleep either occurs specifically under conditions of starvation, or is a general response to nutrient deprivation. To differentiate between these two possibilities we measured sleep in flies selected for desiccation resistance. There was no difference in the sleep duration between DR and F_DR_ flies (Fig 3 and S3 Fig). In agreement with previous findings, all three SR lines slept longer than F_SR_ controls, but no DR line slept longer than its respective F_DR_ control. This confirms that evolutionary selection for SR, but not DR, results in prolonged sleep (Fig 3 and S3 Fig). Sleep can be differentiated from lethargy or hyperactivity by measuring the amount of activity exhibited when an animal is awake [26]. We measured beam crossings per waking minute to infer waking activity in DR flies to determine if they conserve energy by reducing activity, rather than by extending sleep. Waking activity was reduced in all three DR lines compared to F_DR_ controls, while waking activity was not changed (Group A and B) or reduced (Group C) in SR files (Fig 3C and S3 Fig). Therefore, selection for DR does not result in prolonged sleep, but does reduce activity, providing evidence for distinct energy conservation strategies in response to starvation or desiccation conditions.

### DR phenotypes are not due to starvation during selection

**Fig 4:**
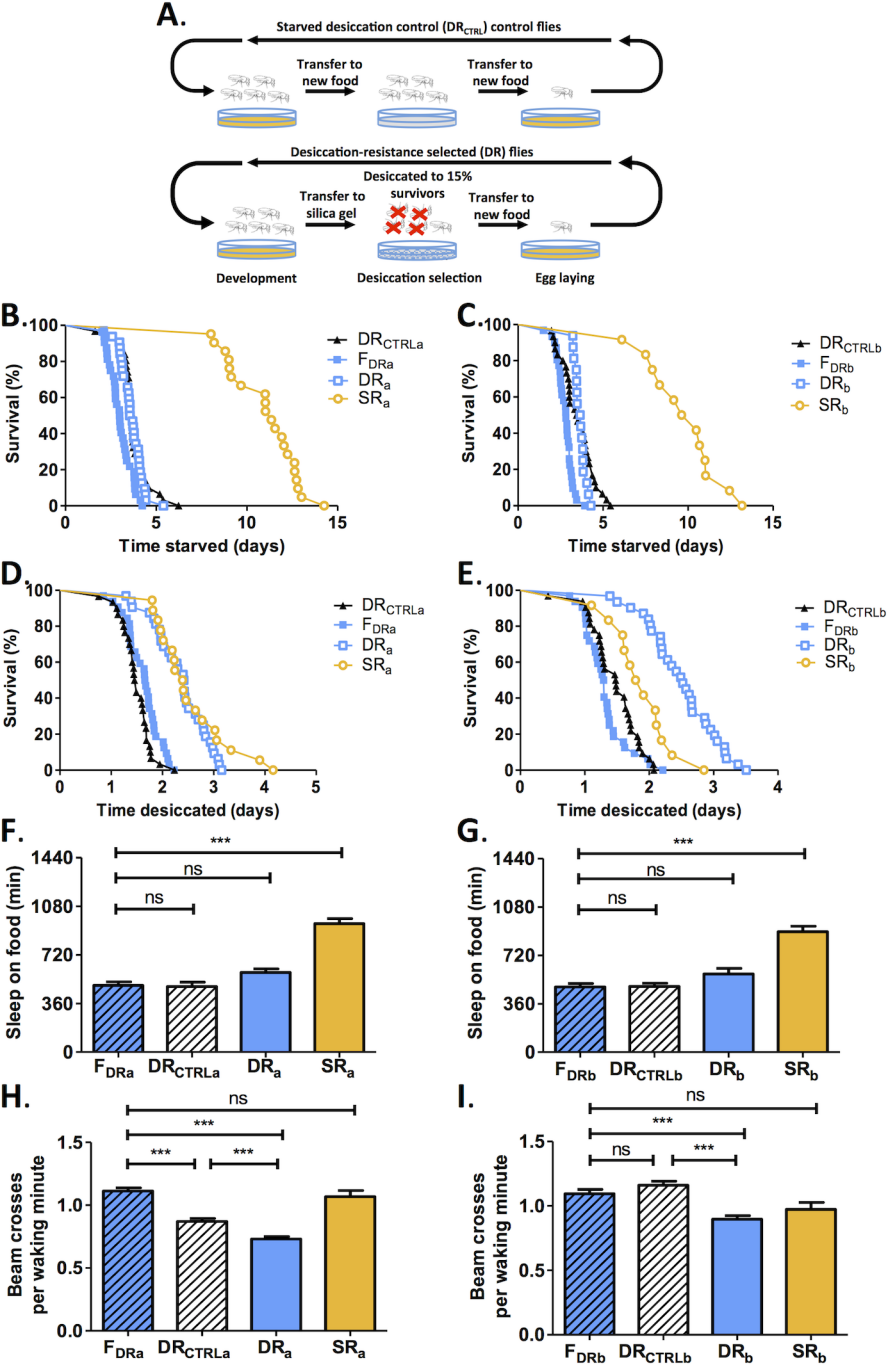
Resistance to nutrient deprivation in DR is not confounded by starvation during selection. A) Schematic of selection process for yoked-control flies (DR_CTRLa_ and DR_CTRLb_) that were starved during the selection period for DR flies. Of the three groups originally generated, only two remain. B, C) Survival of DR_CTRLa_ and DR_CTRLb_ flies does not differ from respective F_DR_ controls under starvation conditions. D, E) Survival of DR_CTRLa_ and DR_CTRLb_ flies does not differ from respective F_DR_ controls under desiccation conditions. F, G) Sleep is not increased in the DR_CTRLa_ and DR_CTRLb_ flies compared to their respective F_DR_ controls or DR selected experimental groups. Sleep is significantly less than respective SR selected flies. H) Beam crossings per waking minute, an meausre of activity while awake, were reduced in DR_CTRLa_ flies compared to F_DRa_ controls, and was significantly greater than SR_a_ selected flies. I) Beam crossings per waking minute did not differ between DR_CTRLb_ and F_DRb_ flies and was significantly greater than both DR_b_ and SR_b_ selected lines. *** denotes P<0.001.

The selection protocol used to generate DR flies creates a state of both food and water deprivation, raising the possibility that resistance to nutrient deprivation and the altered activity levels of DR flies are due to starvation. To account for this possibility we assayed yoked-control flies (DR_CTRL_) that were starved for the period that DR flies were desiccated throughout DR selection (Fig 4A). Only two of the three originally selected DR_CTRL_ groups remain. Survival under starvation conditions (Fig 4B and 4C) and desiccation conditions (Fig 4D and 4E) did not differ between DR_CTRL_ flies or F_DR_ control flies, suggesting that the resistance to nutrient deprivation observed in DR flies results from desiccation selection specifically. Flies from the SR_a_ and SR_b_ groups survived significantly longer than their F_DR_ and DR_CTRL_ controls. This indicates that the relatively short starvation selection time used for DR selection (~3–4 days) is insufficient to confer changes in starvation resistance (Fig 4B and 4C). Further, DR_a_ and DR_b_ group flies survived longer under desiccation conditions than their DR_CTRL_ flies, confirming that survival under desiccation conditions in DR flies is not due to starvation during the selection procedure.

To determine whether starvation during desiccation impacted behavior, we tested DR_CTRL_ flies for sleep and waking activity. Sleep did not differ between F_DR_ and DR_CTRL_ flies, indicating that the selection period was not long enough do induce the increased sleep phenotype observed in all three SR groups (Fig 4F and 4G). Beam crossings per waking minute were reduced in the DR_CTRLa_ control flies compared to F_DRa_ control flies, though not to the levels of the DR_a_ flies, suggesting that starvation partially contributes to the reduced waking activity for Group A (Fig 4H). Conversely, no differences in waking activity, measured by beam crossings per waking minute, was observed between F_DRb_ and DR_CTRLb_ control flies, suggesting that the reduced activity of the DR_b_ flies is not due to starvation during the desiccation selection process (Fig 4I). Taken together, these results fortify the notion that selection under conditions of nutrient deprivation results in differences in survival and behavior that are directly dependent on that water or food loss during the selection process.

### Sleep provides an adaptive response to prolonged starvation

**Fig 5:**
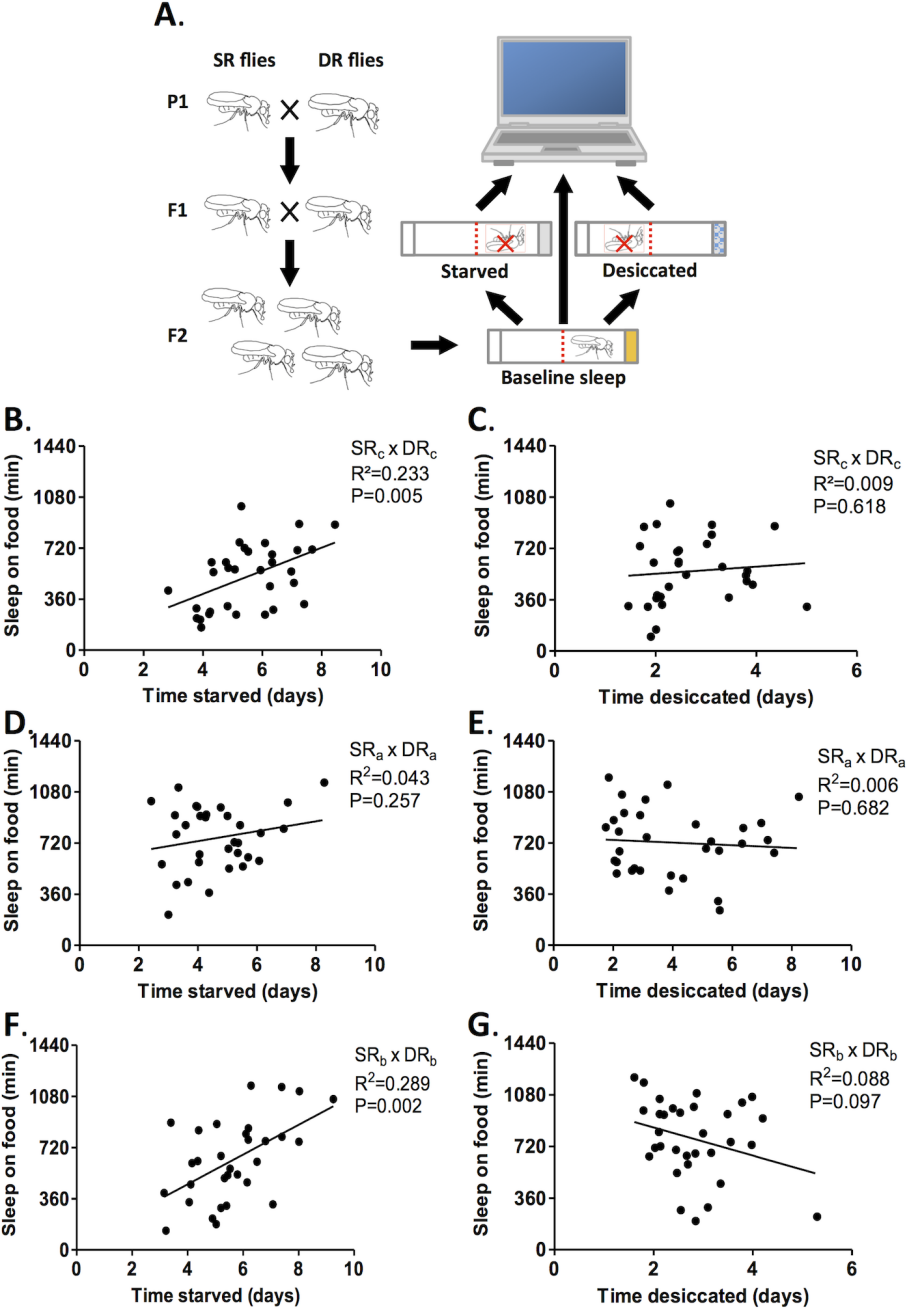
Sleep enhances starvation resistance in SR-DR hybrid flies. A) Schematic of behavioral analysis. F_2_ progeny were generated by crossing SR and DR parental lines. Individuals were then tested for sleep on food over 24hrs, followed by longevity under starvation or desiccation conditions. B) Correlation analysis for SR_c_-DR_c_ F_2_ hybrids reveals a correlation between sleep duration on food and starvation resistance (N = 32 for starved; P<0.01; R^2^ = 0.482). C) No correlation is observed between desiccation resistance and sleep duration on food in SR_c_-DR_c_ F_2_ hybrid flies (N = 30 for desiccated; P>0.05; R^2^ = 0.01). D, E) No correlation between sleep duration and longevity was observed in flies from SR_a_-DR_a_ crosses for either starvation or desiccation (N = 32; P>0.05; R^2^ = 0.043; N = 32; P>0.05; R^2^ = 0.006). F, G) Sleep duration was correlated with starvation resistance in SR_b_-DR_b_ F_2_ hybrid flies (N = 31; P<0.05; R^2^ = 0.289), while no correlation was observed between sleep duration and desiccation resistance (N = 32; P>0.05; R^2^ = 0.068).

The enhanced sleep duration of SR flies raises the possibility that extended sleep is advantageous under conditions of chronic starvation, but not desiccation. To directly test this hypothesis we generated F_2_ hybrids between SR and DR flies. Individual F_2_ flies were tested for sleep duration on food, then transferred to starvation or desiccation conditions, and the relationship between sleep on food and resistance to nutrient deprivation was determined (Fig 5A). A significant positive correlation between sleep and starvation resistance was observed between SR_b_-DR_b_ and SR_c_-DR_c_ populations, suggesting that prolonged sleep is adaptive in food-deprived conditions (Fig 5B and 5F). However, there was no correlation in SR_a_-DR_a_ hybrids, raising the possibility that the advantageous effects of sleep in response to starvation are more dependent on genetic background and evolutionary history (Fig 5D). No relationship was observed between sleep and desiccation resistance for any of the pairings tested (Fig 5C, 5E and 5G). Therefore, prolonged sleep appears to explain up to 30% of the resistance to starvation. Taken together with the prolonged sleep of SR flies, these findings support the notion that adaptations in response to starvation and desiccation conditions result in distinct behavioral and physiological alterations.

## Discussion

Adapting to changes in water and food availability is a central challenge for survival. Animals have developed diverse physiological and behavioral traits to cope with both chronic and acute nutrient shortage [30]. Three primary methods for survival in the absence of nutrients are conservation of energy, elevated accumulation of energy stores, and increased tolerance to energy depletion [31]. Previous studies have indicated developmental, behavioral, and metabolic changes that are associated with starvation resistance [17,24,32]. Desiccation resistance has been linked to increased glycogen stores and changes in cuticular hydrocarbons that prevent water loss [33]. Total carbohydrate content and an increase in hemolymph volume has also been associated with desiccation resistance, suggesting that diverse physiological changes underlie the evolution of this process [16,23,32,34]. Further, a previous study indicated that exposure to chronic stressors, including mechanical and light stimulation, result in a reduction of triglyceride and glycogen stores, raising the possibility that energy stores are generally protective against environmental stressors [35]. Therefore, adaptation to desiccation or starvation conditions likely involves complex interactions between developmental, physiological, and behavioral traits.

In this study, we examine the role of energy conservation through changes in sleep, physiology, and activity in starvation and desiccation resistance. We have utilized experimental evolution to directly investigate the effects of selection for starvation and desiccation resistance on metabolic function and behavior. We identify differences in metabolic function, sleep, and activity response to nutrient deprivation between flies selected for starvation and desiccation resistance, suggesting specialized adaptations to SR or DR conditions. Previous studies examining DR selected flies have identified a generalized resistance to stressors, including chemical stress, heat stress, and radiation stress [16,17]. Our findings suggest that the B and C groups of DR flies are less susceptible to starvation, while starvation resistance does not differ in DR_a_ flies when compared to their controls. None of the DR groups gained resistance to heat stress. These findings highlight how multiple mechanisms likely underlie the evolution of DR, with at least some of these mechanisms being conducive to starvation resistance.

A comparison of energy stores between starvation and desiccation resistant fly lines reveals significant differences between two of the three groups, suggesting distinct survival-promoting mechanisms in response to starvation and desiccation conditions. Selection for starvation resistance increases triglyceride levels in all three groups, while selection for desiccation resistance results in increased glycogen stores in all three groups. Triglycerides are richer in energy compared to glycogen, and mutants with enhanced triglyceride stores have increased SR, suggesting that these promote long-term survival in the absence of food [36]. Glycogen appears to be involved in water binding, allowing animals to increase water weight, making it the more suitable energy store under desiccation conditions [37]. Interestingly, SR_a_ flies had increases in both glycogen and triglyceride levels and were resistant to both starvation and desiccation, fortifying the notion that triglyceride stores promote starvation resistance, while glycogen stores promote desiccation resistance. A number of previous studies have linked starvation and desiccation to enhanced lipid and glycogen levels. However, another study found no relationship between lipid levels and starvation resistance, raising the notion that multiple mechanisms are available for inducing starvation resistance [16,38].

Behavioral changes, including reduced movement and increased sleep duration, may conserve energy and prolong survival during nutrient deprivation. We had previously shown that the SR flies sleep longer than controls, but it was not clear whether this phenotype was a specific response to starvation or a more general response to stress [24]. Two lines of evidence suggest that prolonged sleep is not a generalized adaptation to stress. First, no increase in sleep is observed in desiccation resistant flies, suggesting functional differences between starvation and desiccation resistance. Second, neither selection for desiccation nor starvation resistance affects survival in response to high-heat stress. The findings that SR flies have higher triglyceride stores raise the possibility that triglycerides (fat storage) modulate sleep. However, we previously rescued the body size and triglyceride levels of SR flies by removing larvae from food prior to pupation and found no effect on sleep [24]. Further, we report increased body size of DR flies that sleep normally compared to controls, suggesting that the enhanced sleep in SR flies is independent of energy stores or body size. Therefore, the sleep increase in SR flies is likely due to changes in genetic factors that regulate behavior.

Nutrient availability during development potently affects adult behavior and physiology [39]. It is therefore likely that selection for resistance to nutrient deprivation during the larval or adult state have effects throughout the animal’s life cycle. In this study, both starvation resistant and desiccation resistant larvae were raised on standard fly food. However, selection for resistance to poor food quality during larval development results in reduced larval foraging activity that is influenced by polymorphisms in the *foraging* locus [40]. Further, larvae raised in nutrient poor conditions display many phenotypes associated with reduced food quality, including reduced adult size and prolonged development [40]. Therefore, selection for resistance to nutrient deprivation at the adult or larval stage appears to reduce foraging, although it is unclear whether shared genetic mechanism are involved in these processes. The increased body size of SR flies is, at least partially, due to prolonged larval development, raising the possibility that the starvation and desiccation phenotypes observed may be affected by nutrient availability in the larval stage (Reynolds and Gibbs, Personal Communication). The robust difference of both SR and DR populations to nutrient deprivation may provide a model for investigating the contributions of larval development to these processes.

Multiple studies link total activity to water loss due to increased respiration [33,41], raising the possibility that reduced activity promotes desiccation resistance through decreased respiration. It has been previously reported that flies selected for starvation and desiccation resistance have reduced activity that is uncoupled from respiration, suggesting these two are separable [17,42]. Our findings show reduced activity in SR and DR groups. Reduced waking activity was observed in all three groups of SR selected flies compared to fed controls. All three DR groups displayed significantly reduced activity, or at least trended towards this phenotype, compared to non-selected F_DR_ control flies. Therefore, independent mechanisms appear to have evolved to reduce total activity, whereby selection for SR results in reduced sleep and waking activity, while selection for DR results in reduced waking activity without affecting sleep. While the reasons for this are unclear, we speculate that there is a greater pressure for reduced activity in SR flies, resulting in multiple adaptive strategies, including increased sleep.

It has previously been proposed that sleep or prolonged immobility allows for energy conservation in the absence of food. For example, many animals enhance their sleep or hibernate during winter periods when food is scarce [12]. We generated F_2_ hybrids from SR and DR flies to directly test the assertion that increased sleep is linked to starvation resistance. We found that sleep on food is correlated with starvation resistance for two of the three hybrid groups tested, while there was no correlation between sleep duration and desiccation resistance. Therefore, these findings provide evidence that sleep represents an adaptive behavior that enhances survival in the absence of food, but not in the absence of water.

In conclusion, we have used experimental evolution to examine the effects of desiccation and starvation selection on metabolism and behavior. Flies selected for desiccation or starvation resistance show differences in energy stores, behavioral response to nutrient deprivation, and sleep duration. Sleep duration is enhanced in flies selected for starvation resistance, but no differences are observed in desiccation resistant flies. Longevity under starvation conditions is linked to sleep, supporting the notion that prolonged sleep represents an adaptive evolutionary response to long-term starvation. Therefore, these findings reveal an evolutionary capacity for outbred flies to adapt to distinct forms of nutrient stress, and establish starvation resistant flies as a model for understanding the evolutionary relationship between sleep and survival under nutrient poor conditions.

## Supporting Information

S1 Fig Nutrient stores in SR and DR Flies. A, B) Triglyceride levels in A and B group flies. Triglyceride levels were elevated in SR_a_ and SR_b_ flies compared to F_SR_ controls. No differences in triglyceride levels were observed between DR_a_ and DR_b_ flies and F_DR_ controls (N = 10 and P<0.0001 for all A groups; N = 20 and P<0.01 for F_SRb_ and SR_b_; N = 10 and P>0.05 for F_DRb_ and DR_b_). C, D) Glycogen levels were increased in both SR_a_ and DR_a_ flies compared to respective controls. No differences in glycogen levels were apparent in SR_b_ flies compared to F_SRb_ controls, while glycogen levels were increased in DR_b_ flies compared to F_DRb_ controls (N = 20 for F_SRa_, SF_a_, and DR_a_ groups; N = 18 for F_DRa_; P<0.001 for F_SRa_ and SR_a_; P = 0.002 for F_DRa_ and DR_a_; N = 10 for F_SRb_; N = 7 for SR_b_; N = 9 for F_DRb_ and DR_b_; P>0.05 for F_SRb_ and SR_b_; P<0.001 for F_DRb_ and DR_b_). E, F) Slight to no differences in free glucose were observed between the lines tested (N = 20 for F_SRa_, SF_a_, and DR_a_ groups; N = 18 for F_DRa_; P<0.05 for all A groups; N = 10 for F_SRb_; N = 7 for SR_b_; N = 9 for F_DRb_ and DR_b_; P>0.05 for all B groups).
(PDF)

S2 Fig Longevity in response to nutrient and thermal stress. Survival of flies placed in activity monitors under starvation, desiccation, and heat stress conditions. A, B) Flies from the SR_a_ and SR_b_ groups survived longer than DR counterparts and both controls under starvation conditions. No differences were observed between DR_a_ flies and controls, while DR_b_ flies survived longer than controls under starvation conditions (N = 16 for all A groups and SR_b_, F_DRb_, and DR_b_; N = 14 for F_SRb_; P<0.001 for F_SRa_ and SR_a_, F_SRb_ and SR_b_, F_DRb_ and DRb; P>0.05 for F_DRa_ and DR_a_). C, D) SR_a_ and DR_a_ flies survive longer than controls under desiccation conditions. DR_b_ flies survived longer than SR_b_ flies and both controls under desiccation conditions (N = 32 for F_SRa_, SR_a_, F_DRa_, and DR_b_; N = 31 for DR_a_ and SR_b_; N = 30 for F_SRb_; N = 29 for F_DRb_; P<0.001 for both F_DR_ vs. DR groups; P<0.01 for F_SRa_ vs. SR_a_; P<0.05 for F_SRb_ vs. SR_b_). E, F) No differences were observed between SR and DR flies under thermal stress conditions (N = 32 for all groups; P>0.05 for all groups).
(PDF)

S3 Fig Sleep and activity phenotypes of SR and DR flies. A, B) The total sleep duration over 24hrs on food is significantly longer in SR_a_ and SR_b_ flies compared to F_SRa_ controls. No differences were observed between DR_a_ and DR_b_ flies and control lines (N = 64 for all groups; P<0.0001 for all groups). C, D) Sleep profiles depicting hourly sleep reveal sleep in SR_a_ and SR_b_ flies is increased during both day and night periods compared to the DR groups and both controls (N = 64 for all groups; P<0.001 for both F_SR_ vs. SR groups; P>0.05 for both F_DR_ vs. DR groups). E, F) Waking activity is reduced in DR flies, but not in SR flies, when compared to controls (N = 64 for all groups; P>0.05 for both F_SR_ vs. SR groups; P = 0<0.001 for both F_DR_ vs. DR groups).
(PDF)

S1 Table Detailed statistical analysis. The number of replicates (N) and statistical values are presented for each figure within the main text. ‘NS’ denotes non-significant differences between experimental group and control. * denotes P<0.05, ** denotes P<0.01, *** denotes P<0.001.
(DOCX)
