# Supplementary figures and images for: Enhanced Sleep Is an Evolutionarily Adaptive Response to Starvation Stress in Drosophila
Source: PLoS One. 2015 Jul 6;10(7):e0131275. doi: 10.1371/journal.pone.0131275 (PMC4493134; doi:10.1371/journal.pone.0131275)

**Figure Suppl 1**

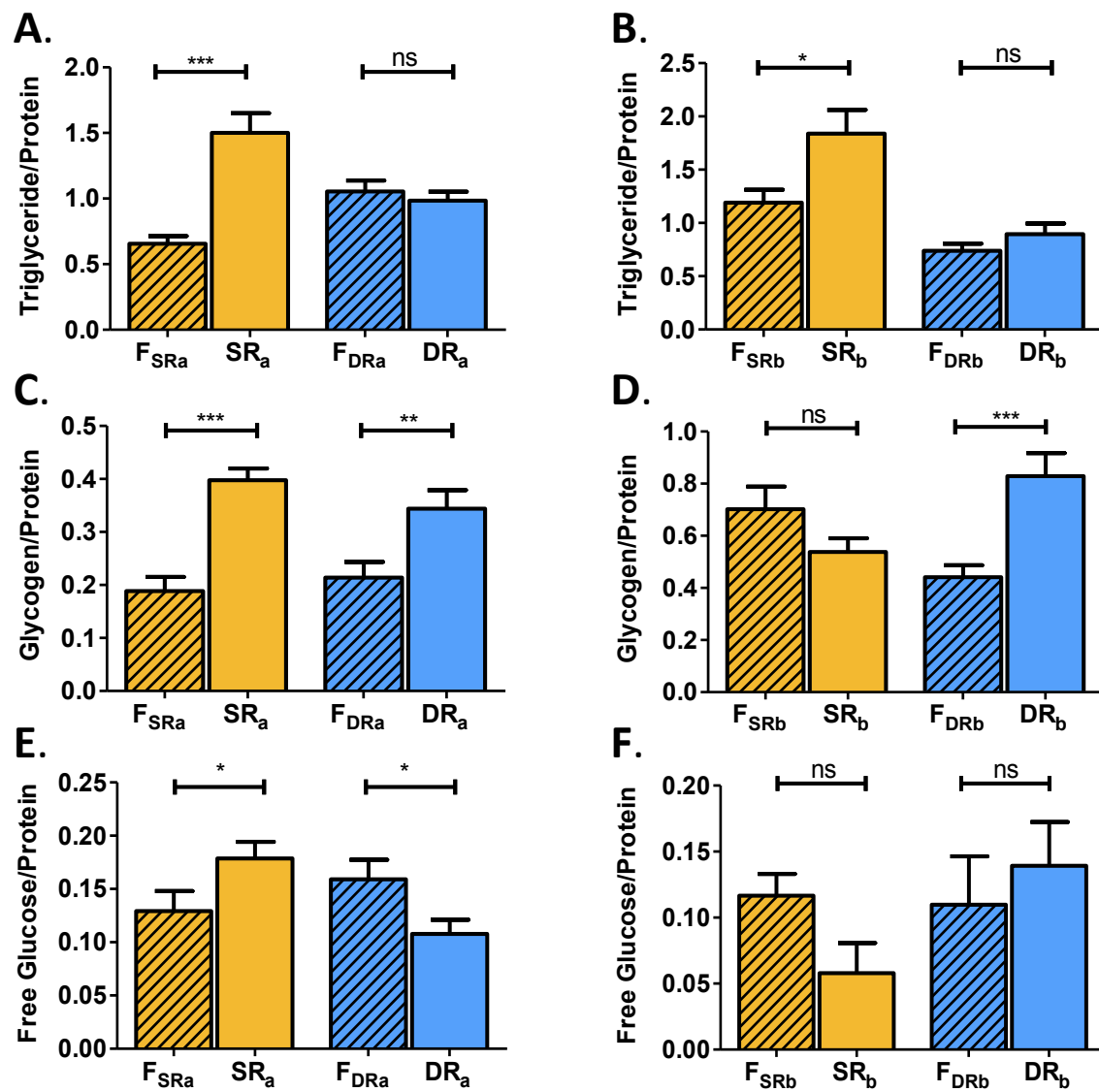

Supplement: S1 Fig — A, B) Triglyceride levels in A and B group flies. Triglyceride levels were elevated in SRa and SRb flies compared to FSR controls. No differences in triglyceride levels were observed between DRa and DRb flies and FDR controls (N = 10 and P<0.0001 for all A groups; N = 20 and P<0.01 for FSRb and SRb; N = 10 and P>0.05 for FDRb and DRb). C, D) Glycogen levels were increased in both SRa and DRa flies compared to respective controls. No differences in glycogen levels were apparent in SRb flies compared to FSRb controls, while glycogen levels were increased in DRb flies compared to FDRb controls (N = 20 for FSRa, SFa, and DRa groups; N = 18 for FDRa; P<0.001 for FSRa and SRa; P = 0.002 for FDRa and DRa; N = 10 for FSRb; N = 7 for SRb; N = 9 for FDRb and DRb; P>0.05 for FSRb and SRb; P<0.001 for FDRb and DRb). E, F) Slight to no differences in free glucose were observed between the lines tested (N = 20 for FSRa, SFa, and DRa groups; N = 18 for FDRa; P<0.05 for all A groups; N = 10 for FSRb; N = 7 for SRb; N = 9 for FDRb and DRb; P>0.05 for all B groups). (PDF) [file pone.0131275.s001.pdf]

**Figure Suppl 2**

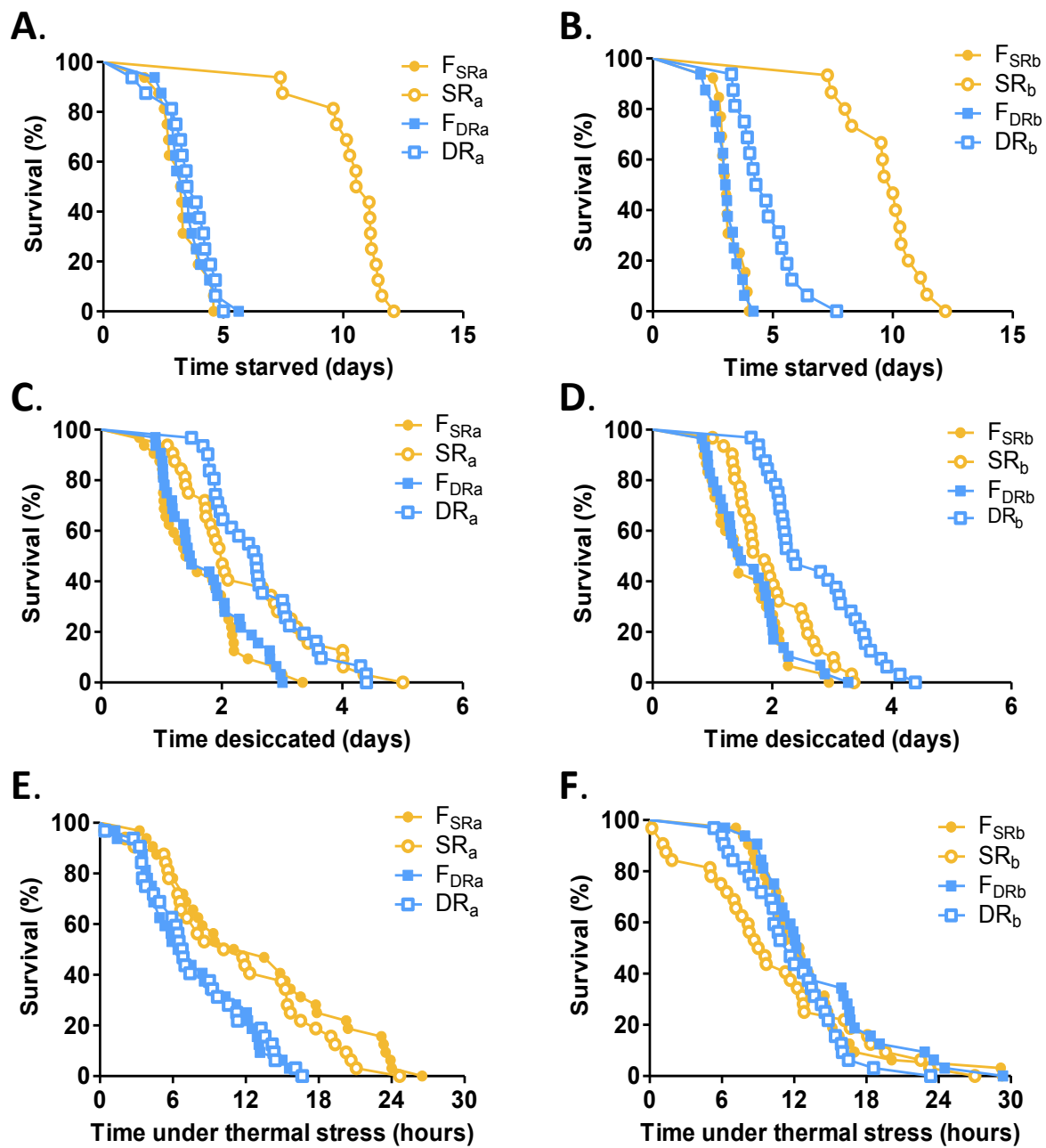

Supplement: S2 Fig — Survival of flies placed in activity monitors under starvation, desiccation, and heat stress conditions. A, B) Flies from the SRa and SRb groups survived longer than DR counterparts and both controls under starvation conditions. No differences were observed between DRa flies and controls, while DRb flies survived longer than controls under starvation conditions (N = 16 for all A groups and SRb, FDRb, and DRb; N = 14 for FSRb; P<0.001 for FSRa and SRa, FSRb and SRb, FDRb and DRb; P>0.05 for FDRa and DRa). C, D) SRa and DRa flies survive longer than controls under desiccation conditions. DRb flies survived longer than SRb flies and both controls under desiccation conditions (N = 32 for FSRa, SRa, FDRa, and DRb; N = 31 for DRa and SRb; N = 30 for FSRb; N = 29 for FDRb; P<0.001 for both FDR vs. DR groups; P<0.01 for FSRa vs. SRa; P<0.05 for FSRb vs. SRb). E, F) No differences were observed between SR and DR flies under thermal stress conditions (N = 32 for all groups; P>0.05 for all groups). (PDF) [file pone.0131275.s002.pdf]

**Figure Suppl 3**

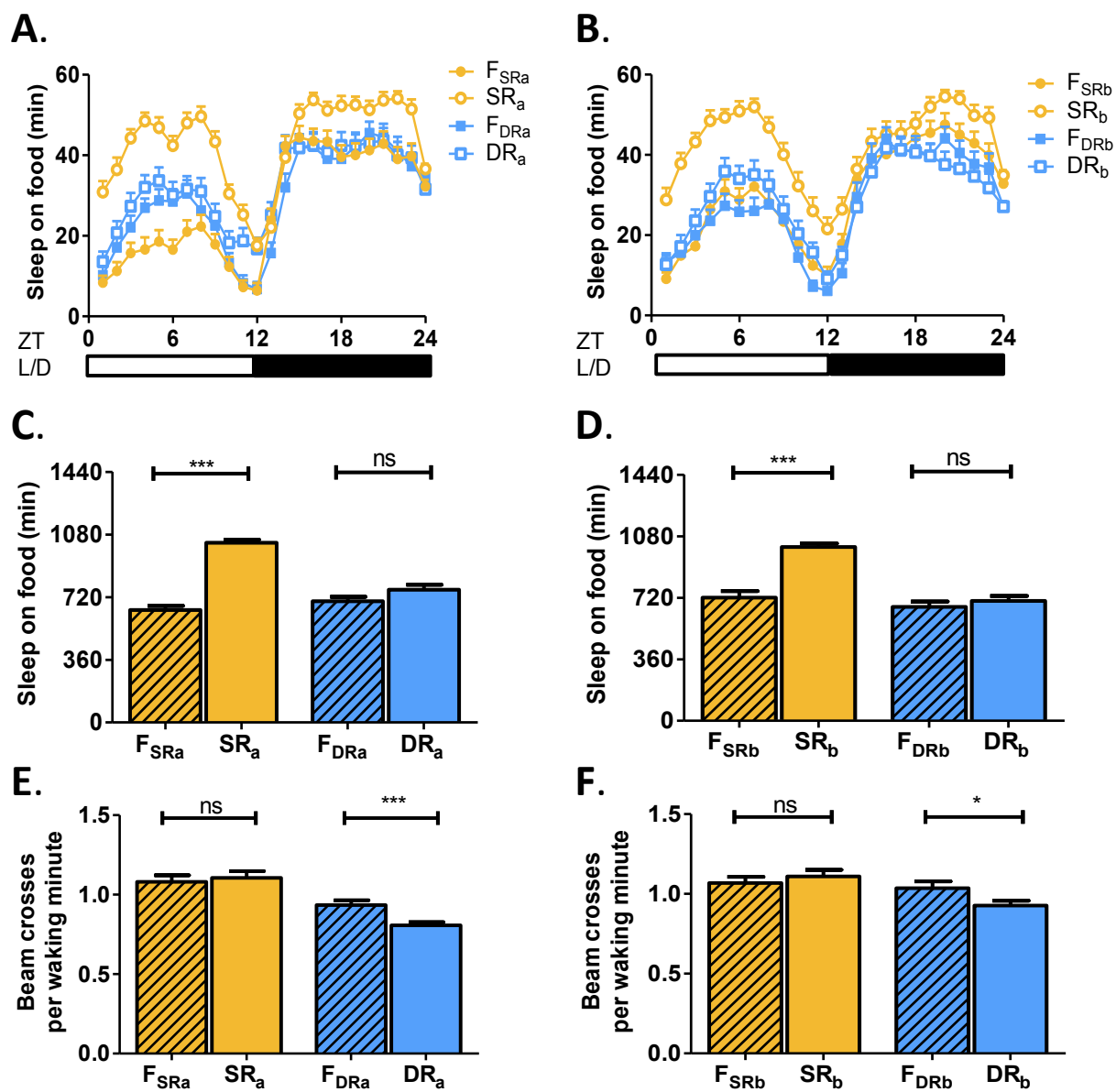

Supplement: S3 Fig — A, B) The total sleep duration over 24hrs on food is significantly longer in SRa and SRb flies compared to FSRa controls. No differences were observed between DRa and DRb flies and control lines (N = 64 for all groups; P<0.0001 for all groups). C, D) Sleep profiles depicting hourly sleep reveal sleep in SRa and SRb flies is increased during both day and night periods compared to the DR groups and both controls (N = 64 for all groups; P<0.001 for both FSR vs. SR groups; P>0.05 for both FDR vs. DR groups). E, F) Waking activity is reduced in DR flies, but not in SR flies, when compared to controls (N = 64 for all groups; P>0.05 for both FSR vs. SR groups; P = 0<0.001 for both FDR vs. DR groups). (PDF) [file pone.0131275.s003.pdf]
