# Supplementary material for: Enhanced Sleep Is an Evolutionarily Adaptive Response to Starvation Stress in Drosophila
Source: PLoS One. 2015 Jul 6;10(7):e0131275. doi: 10.1371/journal.pone.0131275 (PMC4493134; doi:10.1371/journal.pone.0131275)
Supplement: S1 Table — The number of replicates (N) and statistical values are presented for each figure within the main text. ‘NS’ denotes non-significant differences between experimental group and control. * denotes P<0.05, ** denotes P<0.01, *** denotes P<0.001. (DOCX) [file pone.0131275.s004.docx]

| **Figure No.** | **Condition** | **Statics method** | **N** | **Statistics value** | **P-value** |  |
| --- | --- | --- | --- | --- | --- | --- |
| Figure 1C | **GroupC**  Triglyceride | ANOVA | N = 10, 10, 10 and 10 for F_SR_, SR, F_DR_ and DR | F_3,36_ = 6.3 | P = 0.001 | ****** |
|  |  | Planned contrast: F_SR_ vs SR | N = 10, 10 for F_SR_ and SR | t_36_ = -4.3 | P < 0.001 | ******* |
|  |  | Planned contrast: F_DR_ vs DR | N = 10, 10 for F_DR_ and DR | t_36_ = -0.3 | P = 0.799 | **NS** |
| Suppl Fig 1A | **GroupA**  Triglyceride | ANOVA | N = 10, 10, 10 and 10 for F_SR_, SR, F_DR_ and DR | F_3,36_ = 12.9 | P < 0.001 | ******* |
|  |  | Planned contrast: F_SR_ vs SR | N = 10, 10 for F_SR_, SR | t_11.6_ = -5.2 | P < 0.001 | ******* |
|  |  | Planned contrast: F_DR_ vs DR | N = 10, 10 for F_DR_, DR | t_17.4_ = 0.7 | P = 0.519 | **NS** |
| Suppl Fig 1B | **GroupB**  Triglyceride | ANOVA | N = 20, 20, 10 and 10 for F_SR_, SR, F_DR_ and DR | F_3,56_ = 7.8 | P < 0.001 | ******* |
|  |  | Planned contrast: F_SR_ vs SR | N = 20, 20 for F_SR_, SR | t_29.4_ = -2.5 | P = 0.016 | ***** |
|  |  | Planned contrast: F_DR_ vs DR | N = 10, 10 for F_DR_, DR | t_15.7_ = -1.3 | P = 0.212 | **NS** |
| Figure 1D | **GroupC**  Glycogen | ANOVA | N = 10, 10, 10 and 10 for F_SR_, SR, F_DR_ and DR | F_3,36_ = 6.3 | P = 0.001 | ****** |
|  |  | Planned contrast: F_SR_ vs SR | N = 10, 10 for F_SR_ and SR | t_36_ = -0.9 | P = 0.375 | **NS** |
|  |  | Planned contrast: F_DR_ vs DR | N = 10, 10 for F_DR_ and DR | t_36_ = -4.0 | P < 0.001 | ******* |
| Suppl Fig 1C | **GroupA**  Glycogen | ANOVA | N = 20, 20, 18 and 20 for F_SR_, SR, F_DR_ and DR | F_3,74_ = 12.4 | P < 0.001 | ******* |
|  |  | Planned contrast: F_SR_ vs SR | N = 20, 20 for F_SR_, SR | t_74_ = -5.2 | P < 0.001 | ******* |
|  |  | Planned contrast: F_DR_ vs DR | N = 18, 20 for F_DR_, DR | t_74_ = -2.2 | P = 0.002 | ****** |
| Suppl Fig 1D | **GroupB**  Glycogen | ANOVA | N = 10, 7, 9 and 9 for F_SR_, SR, F_DR_ and DR | F_3,31_ = 6.0 | P = 0.002 | ****** |
|  |  | Planned contrast: F_SR_ vs SR | N = 10, 7 for F_SR_, SR | t_31_ = 1.4 | P = 0.183 | **NS** |
|  |  | Planned contrast: F_DR_ vs DR | N = 9, 9 for F_DR_, DR | t_31_ = -4.0 | P < 0.001 | ******* |
| Figure 1E | **GroupC**  Free Glucose | ANOVA | N = 10, 10, 10 and 10 for F_SR_, SR, F_DR_ and DR | F_3,36_ = 0.1 | P = 0.930 | **NS** |
| Suppl Fig 1E | **GroupA**  Free Glucose | ANOVA | N = 20, 20, 18 and 20 for F_SR_, SR, F_DR_ and DR | F_3,74_ = 3.6 | P = 0.017 | ***** |
|  |  | Planned contrast: F_SR_ vs SR | N = 20, 20 for F_SR_, SR | t_74_ = -2.1 | P = 0.037 | ***** |
|  |  | Planned contrast: F_DR_ vs DR | N = 18, 20 for F_DR_, DR | t_74_ = 2.2 | P = 0.035 | ***** |
| Suppl Fig 1F | **GroupB**  Free Glucose | ANOVA | N = 10, 7, 9 and 9 for F_SR_, SR, F_DR_ and DR | F_3,31_ = 1.2 | P = 0.334 | **NS** |
| Figure 2A | **GroupC**  Survival (starvation) | Kaplan-Meier all | N = 16, 16, 16 and 16 for F_SR_, SR, F_DR_ and DR | Log rank: χ^2^ = 48.7, df = 3 | P < 0.001 | ******* |
|  |  | Kaplan-Meier F_SR_ vs SR, Bonferroni correction | N = 16, 16 for F_SR_, SR | Log rank: χ^2^ = 36.6, df = 1 | P < 0.001 | ******* |
|  |  | Kaplan-Meier F_DR_ vs DR, Bonferroni correction | N = 16, 16 for F_DR_ and DR | Log rank: χ^2^ = 0.1, df = 1 | P = 0.717 | **NS** |
| Suppl Fig 2A | **GroupA**  Survival (starvation) | Kaplan-Meier all | N = 16, 16, 16 and 16 for F_SR_, SR, F_DR_ and DR | Log rank: χ^2^ = 48.7, df = 3 | P < 0.001 | ******* |
|  |  | Kaplan-Meier F_SR_ vs SR, Bonferroni correction | N = 16, 16 for F_SR_, SR | Log rank: χ^2^ = 36.6, df = 1 | P < 0.001 | ******* |
|  |  | Kaplan-Meier F_DR_ vs DR, Bonferroni correction | N = 16, 16 for F_DR_, DR | Log rank: χ^2^ = 0.1, df = 1 | P = 0.717 | **NS** |
| Suppl Fig 2B | **GroupB**  Survival (starvation) | Kaplan-Meier all | N = 14, 16, 16 and 16 for F_SR_, SR, F_DR_ and DR | Log rank: χ^2^ = 71.9, df = 3 | P < 0.001 | ******* |
|  |  | Kaplan-Meier F_SR_ vs SR, Bonferroni correction | N = 14, 16 for F_SR_, SR | Log rank: χ^2^ = 35.6, df = 1 | P < 0.001 | ******* |
|  |  | Kaplan-Meier F_DR_ vs DR, Bonferroni correction | N = 16, 16 for F_DR_, DR | Log rank: χ^2^ = 22.1, df = 1 | P < 0.001 | ******* |
| Figure 2B | **GroupC**  Survival (Desiccation) | Kaplan-Meier all | N = 32, 30, 32 and 31 for F_SR_, SR, F_DR_ and DR | Log rank: χ^2^ = 124.0, df = 3 | P < 0.001 | ******* |
|  |  | Kaplan-Meier F_SR_ vs SR, Bonferroni correction | N = 32, 30 for F_SR_, SR | Log rank: χ^2^ = 23.0, df = 1 | P < 0.001 | ******* |
|  |  | Kaplan-Meier F_DR_ vs DR, Bonferroni correction | N = 32, 31 for F_DR_, DR | Log rank: χ^2^ = 65.8, df = 1 | P < 0.001 | ******* |
| Suppl Fig 2C | **GroupA**  Survival (Desiccation) | Kaplan-Meier all | N = 32, 32, 32 and 31 for F_SR_, SR, F_DR_ and DR | Log rank: χ^2^ = 28.1, df = 3 | P < 0.001 | ******* |
|  |  | Kaplan-Meier F_SR_ vs SR, Bonferroni correction | N = 32, 32 for F_SR_, SR | Log rank: χ^2^ = 9.8, df = 1 | P = 0.004 | ****** |
|  |  | Kaplan-Meier F_DR_ vs DR, Bonferroni correction | N = 32, 31 for F_DR_, DR | Log rank: χ^2^ = 17.2, df = 1 | P < 0.001 | ******* |
| Suppl Fig 2D | **GroupB**  Survival (Desiccation) | Kaplan-Meier all | N = 30, 31, 29 and 32 for F_SR_, SR, F_DR_ and DR | Log rank: χ^2^ = 39.3, df = 3 | P < 0.001 | ******* |
|  |  | Kaplan-Meier F_SR_ vs SR, Bonferroni correction | N = 30, 31 for F_SR_, SR | Log rank: χ^2^ = 5.7, df = 1 | P = 0.034 | ***** |
|  |  | Kaplan-Meier F_DR_ vs DR, Bonferroni correction | N = 29, 32 for F_DR_, DR | Log rank: χ^2^ = 27.8, df = 1 | P < 0.001 | ******* |
| Figure 2C | **GroupC**  Survival (Thermal stress) | Kaplan-Meier all | N = 31, 31, 31 and 32 for F_SR_, SR, F_DR_ and DR | Log rank: χ^2^ = 10.4, df = 3 | P = 0.030 | ***** |
|  |  | Kaplan-Meier F_SR_ vs SR, Bonferroni correction | N = 31, 31 for F_SR_, SR | Log rank: χ^2^ = 7.7, df =1 | P = 0.010 (SR show shorter life than FSR) | ***** |
|  |  | Kaplan-Meier F_DR_ vs DR, Bonferroni correction | N = 31, 32 for F_DR_, DR | Log rank: χ^2^ = 0.0, df = 1 | P = 0.834 | **NS** |
| Suppl Fig 2E | **GroupA**  Survival (Thermal stress) | Kaplan-Meier all | N = 32, 32, 32 and 32 for F_SR_, SR, F_DR_ and DR | Log rank: χ^2^ = 23.2, df = 3 | P < 0.001 | ******* |
|  |  | Kaplan-Meier F_SR_ vs SR, Bonferroni correction | N = 32, 32 for F_SR_, SR | Log rank: χ^2^ = 1.2, df = 1 | P = 0.530 | **NS** |
|  |  | Kaplan-Meier F_DR_ vs DR, Bonferroni correction | N = 32, 32 for F_DR_, DR | Log rank: χ^2^ = 0.1, df = 1 | P = 0.745 | **NS** |
| Suppl Fig 2F | **GroupB**  Survival (Thermal stress) | Kaplan-Meier all | N = 32, 32, 32 and 32 for F_SR_, SR, F_DR_ and DR | Log rank: χ^2^ = 4.4, df = 3 | P = 0.219 | **NS** |
| Figure 3B | **GroupC**  % Sleep | ANOVA | N = 64, 64, 64, 64 for F_SR_, SR, F_DR_ and DR | F_3, 252_ = 21.3, df = 3 | P < 0.001 | ******* |
|  |  | Planned contrast: F_SR_ vs SR | N = 64, 64 for F_SR_, SR | t_252_ = -5.9 | P < 0.001 | ******* |
|  |  | Planned contrast: F_DR_ vs DR | N = 64, 64 for F_DR_, DR | t_252_ = -0.6 | P = 0.567 | **NS** |
| Suppl Fig 3C | **GroupA**  % Sleep | ANOVA | N = 64, 64, 64, 63 for F_SR_, SR, F_DR_ and DR | F_3, 251_ = 49.6, df = 3 | P < 0.001 | ******* |
|  |  | Planned contrast: F_SR_ vs SR | N = 64, 64 for F_SR_, SR | t_117.5_ = -12.9 | P < 0.001 | ******* |
|  |  | Planned contrast: F_DR_ vs DR | N= 64, 63 for F_DR_, DR | t_123.5_ = -1.7 | P = 0.089 | **NS** |
| Suppl Fig 3D | **GroupB**  % Sleep | ANOVA | N = 60, 64, 62, 64 for F_SR_, SR, F_DR_ and DR | F_3, 246_ = 29.3, df = 3 | P < 0.001 | ******* |
|  |  | Planned contrast: F_SR_ vs SR | N = 60, 64 for F_SR_, SR | t_92.9_ = -6.9 | P < 0.001 | ******* |
|  |  | Planned contrast: F_DR_ vs DR | N = 62, 64 for F_DR_, DR | t_123.4_ = -0.8 | P = 0.423 | **NS** |
| Figure 3C | **GroupC**  Waking activity | ANOVA | N = 64, 64, 64, 64 for F_SR_, SR, F_DR_ and DR | F_3, 252_ = 26.6, df = 3 | P < 0.001 | ******* |
|  |  | Planned contrast: F_SR_ vs SR | N = 64, 64 for F_SR_, SR | t_105.1_ = 7.4 | P < 0.001 | ******* |
|  |  | Planned contrast: F_DR_ vs DR | N = 64, 64 for F_DR_, DR | t_122.0_ = 4.1 | P < 0.001 | ******* |
| Suppl Fig 3E | **GroupA**  Waking activity | ANOVA | N = 64, 64, 64, 63 for F_SR_, SR, F_DR_ and DR | F_3, 251_ = 15.8, df = 3 | P < 0.001 | ******* |
|  |  | Planned contrast: F_SR_ vs SR | N = 64, 64 for F_SR_, SR | t_125.9_ = -0.4 | P = 0.681 | **NS** |
|  |  | Planned contrast: F_DR_ vs DR | N= 64, 63 for F_DR_, DR | t_110.3_ = 3.6 | P < 0.001 | ******* |
| Suppl Fig 3F | **GroupB**  Waking activity | ANOVA | N = 64, 64, 64, 63 for F_SR_, SR, F_DR_ and DR | F_3, 246_ = 4.2, df = 3 | P = 0.006 | ****** |
|  |  | Planned contrast: F_SR_ vs SR | N = 64, 64 for F_SR_, SR | t_246_ = -0.9 | P = 0.387 | **NS** |
|  |  | Planned contrast: F_DR_ vs DR | N = 64, 63 for F_DR_, DR | t_246_ = 2.2 | P = 0.029 | ***** |
| Figure 4B | **GroupA**  Survival (Starvation) | Kaplan-Meier all | N = 32, 31, 32 and 21 for F_DR_, DR_CTRL_, DR and SR | Log rank: χ^2^ = 87.7, df = 3 | P < 0.001 | ******* |
|  |  | Kaplan-Meier F_DR_ vs DR_CTRL_, Bonferroni correction | N = 32, 31 for F_DR_, DR_CTRL_ | Log rank: χ^2^ = 11.8, df = 1 | P < 0.01 | ****** |
|  |  | Kaplan-Meier F_DR_ vs DR, Bonferroni correction | N = 32, 32 for F_DR_, DR | Log rank: χ^2^ = 11.4, df = 1 | P < 0.01 | ****** |
|  |  | Kaplan-Meier DR_CTRL_ vs DR, Bonferroni correction | N = 31, 32 for DR_CTRL_, DR | Log rank: χ^2^ = 0.2, df = 1 | P > 0.05 | **NS** |
|  |  | Kaplan-Meier DR vs SR, Bonferroni correction | N = 32, 21 for DR, SR | Log rank: χ^2^ = 53.3, df = 1 | P < 0.001 | ******* |
| Figure 4C | **GroupB**  Survival (Starvation) | Kaplan-Meier all | N = 31, 30, 30 and 12 for F_DR_, DR_CTRL_, DR and SR | Log rank: χ^2^ = 96.7, df = 3 | P < 0.001 | ******* |
|  |  | Kaplan-Meier F_DR_ vs DR_CTRL_, Bonferroni correction | N = 31, 30 for F_DR_, DR_CTRL_ | Log rank: χ^2^ = 17.7, df = 1 | P < 0.001 | ******* |
|  |  | Kaplan-Meier F_DR_ vs DR, Bonferroni correction | N = 31, 30 for F_DR_, DR | Log rank: χ^2^ = 53.8, df = 1 | P < 0.001 | ******* |
|  |  | Kaplan-Meier DR_CTRL_ vs DR, Bonferroni correction | N = 30, 30 for DR_CTRL_, DR | Log rank: χ^2^ = 0.1, df = 1 | P > 0.05 | **NS** |
|  |  | Kaplan-Meier DR vs SR, Bonferroni correction | N = 30, 12 for DR, SR | Log rank: χ^2^ = 32.9, df = 1 | P < 0.001 | ******* |
| Figure 4D | **GroupA**  Survival (Desiccated) | Kaplan-Meier all | N = 32, 30, 32 and 19 for F_DR_, DR_CTRL_, DR and SR | Log rank: χ^2^ = 89.7, df = 3 | P < 0.001 | ******* |
|  |  | Kaplan-Meier F_DR_ vs DR_CTRL_, Bonferroni correction | N = 32, 30 for F_DR_, DR_CTRL_ | Log rank: χ^2^ = 3.2, df = 1 | P > 0.05 | **NS** |
|  |  | Kaplan-Meier F_DR_ vs DR, Bonferroni correction | N = 32, 32 for F_DR_, DR | Log rank: χ^2^ = 39.1, df = 1 | P < 0.001 | ******* |
|  |  | Kaplan-Meier DR_CTRL_ vs DR, Bonferroni correction | N = 30, 32 for DR_CTRL_, DR | Log rank: χ^2^ = 48.3, df = 1 | P < 0.001 | ******* |
|  |  | Kaplan-Meier DR vs SR, Bonferroni correction | N = 32, 19 for DR, SR | Log rank: χ^2^ = 1.9, df = 1 | P > 0.05 | **NS** |
| Figure 4E | **GroupB**  Survival (Desiccated) | Kaplan-Meier all | N = 32, 32, 31 and 12 for F_DR_, DR_CTRL_, DR and SR | Log rank: χ^2^ = 79.9, df = 3 | P < 0.001 | ******* |
|  |  | Kaplan-Meier F_DR_ vs DR_CTRL_, Bonferroni correction | N = 32, 32 for F_DR_, DR_CTRL_ | Log rank: χ^2^ = 2.1, df = 1 | P > 0.05 | **NS** |
|  |  | Kaplan-Meier F_DR_ vs DR, Bonferroni correction | N = 32, 31 for F_DR_, DR | Log rank: χ^2^ = 59.5, df = 1 | P < 0.001 | ******* |
|  |  | Kaplan-Meier DR_CTRL_ vs DR, Bonferroni correction | N = 32, 31 for DR_CTRL_, DR | Log rank: χ^2^ = 55.2, df = 1 | P < 0.001 | ******* |
|  |  | Kaplan-Meier DR vs SR, Bonferroni correction | N = 31, 12 for DR, SR | Log rank: χ^2^ = 13.7, df = 1 | P < 0.001 | ******* |
| Figure 4F | **GroupA**  % Sleep | Kruskal-Wallis | N = 61, 59, 61 and 25 for F_DR_, DR_CTRL_, DR and SR | χ^2^ = 53.5, df = 3 | P < 0.001 | ******* |
|  |  | Mann-Whitney F_DR_ vs DR_CTRL_, Bonferroni correction | N = 61, 59 for F_DR_, DR_CTRL_ | U = 1787, df = 1 | P > 0.05 | **NS** |
|  |  | Mann-Whitney F_DR_ vs DR, Bonferroni correction | N = 61, 61 for F_DR_, DR | U = 1339, df = 1 | P > 0.05 | **NS** |
|  |  | Mann-Whitney F_DR_ vs SR, Bonferroni correction | N = 61, 25 for F_DR_, SR | U = 89, df = 1 | P < 0.001 | ******* |
| Figure 4G | **GroupB**  % Sleep | Kruskal-Wallis | N = 64, 63, 61 and 24 for F_DR_, DR_CTRL_, DR and SR | χ^2^ = 38.4, df = 3 | P < 0.001 | ******* |
|  |  | Mann-Whitney F_DR_ vs DR_CTRL_, Bonferroni correction | N = 64, 63 for F_DR_, DR_CTRL_ | U = 1957, df = 1 | P > 0.05 | **NS** |
|  |  | Mann-Whitney F_DR_ vs DR, Bonferroni correction | N = 64, 61 for F_DR_, DR | U = 1706, df = 1 | P > 0.05 | **NS** |
|  |  | Mann-Whitney F_DR_ vs SR, Bonferroni correction | N = 64, 24 for F_DR_, SR | U = 117, df = 1 | P < 0.001 | ******* |
|  |  |  |  |  |  |  |
|  |  |  |  |  |  |  |
| Figure 4H | **GroupA**  Waking activity | Kruskal-Wallis | N = 61, 59, 61 and 41 for F_DR_, DR_CTRL_, DR and SR | χ^2^ = 90.5, df = 3 | P < 0.001 | ******* |
|  |  | Mann-Whitney F_DR_ vs DR_CTRL_, Bonferroni correction | N = 61, 63 for F_DR_, DR_CTRL_ | U = 666, df = 1 | P < 0.001 | ******* |
|  |  | Mann-Whitney F_DR_ vs DR, Bonferroni correction | N = 61, 61 for F_DR_, DR | U = 246, df = 1 | P < 0.001 | ******* |
|  |  | Mann-Whitney F_DR_ vs SR, Bonferroni correction | N = 61, 41 for F_DR_, SR | U = 1070, df = 1 | P > 0.05 | **NS** |
|  |  | Mann-Whitney DR_CTRL_ vs DR, Bonferroni correction | N = 59, 61 for DR_CTRL_, DR | U = 943, df = 1 | P < 0.001 | ******* |
| Figure 4I | **GroupB**  Waking activity | Kruskal-Wallis | N = 64, 63, 61 and 24 for F_DR_, DR_CTRL_, DR and SR | χ^2^ = 38.4, df = 3 | P < 0.001 | ******* |
|  |  | Mann-Whitney F_DR_ vs DR_CTRL_, Bonferroni correction | N = 64, 63 for F_DR_, DR_CTRL_ | U = 1642, df = 1 | P > 0.05 | **NS** |
|  |  | Mann-Whitney F_DR_ vs DR, Bonferroni correction | N = 64, 61 for F_DR_, DR | U = 1072, df = 1 | P < 0.001 | ******* |
|  |  | Mann-Whitney F_DR_ vs SR, Bonferroni correction | N = 64, 24 for F_DR_, SR | U = 536, df = 1 | P > 0.05 | **NS** |
|  |  | Mann-Whitney DR_CTRL_ vs DR, Bonferroni correction | N = 63, 61 for DR_CTRL_, DR | U = 748, df = 1 | P < 0.001 | ******* |

Supplemental Table1, Statistics Summary. *: P < 0.05, **: P < 0.01, ***: P < 0.001, NS: not significant.
